# Spatial Distribution of *Dicrocoelium* in the Himalayan Ranges: Potential Impacts of Ecological Niches and Climatic Variables

**DOI:** 10.1007/s11686-022-00634-1

**Published:** 2022-11-22

**Authors:** Muhammad Asim Khan, Kiran Afshan, Neil D. Sargison, Martha Betson, Sabika Firasat, Umer Chaudhry

**Affiliations:** 1 Department of Zoology, Faculty of Biological Sciences, Quaid-I-Azam University, Islamabad, 45320 Pakistan; 2 School of Veterinary Medicine, Faculty of Health and Medical Sciences, University of Surrey, Daphne Jackson Rd, Guildford, GU2 7AL UK; 3 Royal (Dick) School of Veterinary Studies, Easter Bush Veterinary Centre, University of Edinburgh, Edinburgh, EH25 9RG UK

**Keywords:** Dicrocoeliosis, Himalayas range, Ecological niches, Climatic variables, Sheep, Goats

## Abstract

**Purpose:**

Dicrocoeliosis can be an important cause of production loss in ruminants due to the cost of liver condemnation at slaughter. The aim of the present study was to determine the prevalence of *Dicrocoelium* infection and to predict the ecological niches and climatic variables that support dicrocoeliosis in the Himalayan ranges of Pakistan.

**Methods and Results:**

*Dicrocoelium* was detected in 33 of 381 liver samples and 238 of 6060 blood samples taken from sheep and goat herds in the area. The prevalence of dicrocoeliosis was higher in sheep than in goats and highest in females aged more than 3 years. An environmental risk map was created to predict active zones of transmission and showed the highest probability values in central parts of the Chitral district in the northwest of Pakistan. Climatic variables of the mean monthly diurnal temperature range (Bio2), annual precipitation (Bio12), and normalised difference vegetation index (NDVI) were found to be significantly (*p* < 0.05) associated with the presence of *Dicrocoelium* infection.

**Conclusion:**

Together, the findings of this study demonstrate the most suitable ecological niches and climatic variables influencing the risk of dicrocoeliosis in the Himalayan ranges of Pakistan. The methods and results could be used as a reference to inform the control of dicrocoeliosis in the region.

**Supplementary Information:**

The online version contains supplementary material available at 10.1007/s11686-022-00634-1.

## Introduction

Dicrocoeliosis is an important parasitic disease caused by three species of the genus *Dicrocoelium*, namely *Dicrocoelium dendriticum*, *Dicrocoelium hospes* and *Dicrocoelium chinensis* [1]. Among these, *D. dendriticum* is the most common and is distributed throughout Europe, Asia, North and South America, Australia, and North Africa. The other species have limited distribution and are present in Asia, Africa and some parts of Europe [2]. *Dicrocoelium* can infect the bile ducts of a variety of wild and domesticated mammals. Dicrocoeliosis causes overt economic loss due to the condemnation of livers with cholangitis from slaughtered animals at meat inspection [3]. Clinical signs of poor food intake, ill thrift, poor milk production, alteration in faecal consistency, photosensitisation and anaemia have been described in animals with high burdens [4, 5], and subclinical infection might cause reduced growth, although this is seldom measured.

*Dicrocoelium* has an exceptional life cycle that can take at least 6 months to complete. Within the same geographical location, several species of land snails and ants can be involved as first and second intermediate hosts, respectively [6]. Adult flukes are found in the bile ducts of their definitive herbivorous hosts. Eggs containing fully developed miracidia are shed in faeces and must be ingested by the snails before hatching and undergoing asexual replication and development into cercariae, which are shed by the snails and then eaten by ants. One cercaria migrates into the head of the ant and associates with the suboesophageal ganglion, while up to about 50 encyst in the gaster as metacercariae [7]. The larval stage that develops in the ant’s head alters its behaviour, making it cling to herbage and increasing the probability of its being eaten by a definitive host. Following the encystment of the metacercariae, larval flukes migrate to the liver via the biliary tree and develop into adults [4].

Several studies have described the prevalence of *Dicrocoelium* in endemic regions; 4.8 and 11% in Iran [8, 9], between 5 and 30% in Canada [10, 11], 0.7% in China [12] and 22% in Japan [13]. Due to its unique life cycle involving two intermediate hots, *Dicrocoelium* is highly affected by climatic factors. Temperature and humidity influence the survival of eggs containing miracidia and the development of snail and ant intermediate hosts in their respective environmental niches [9, 10]. A seasonal pattern of the probability of infection has been shown in Canadian livestock, with the highest rate in mid-summer followed by an autumn decline (Dempsey, Burg [10].

Due to the association between these environmental factors and the prevalence and geographical distribution of *Dicrocoelium* infection, species distribution models (SDMs) have the potential to determine the spatial pattern of disease and ecological niches supporting infection challenge. SDMs are based on the interaction between species adaptability and key predicting climatic factors informed by humidity, rainfall, temperature and altitude [14–17]. Geographical Information Systems (GIS) and Maximum Entropy (MaxEnt) are the most widely used SDMs in the study of fluke parasites. These models have been used to show the geographical distribution and spatial pattern of fascioliosis or schistosomiasis and their risk factors associated with the ecological niches and climatic conditions [18–22]

*Dicrocoelium* was first identified in the Himalayan ranges of Pakistan by Khan, Afshan [23]. There have been few studies that provide information on the spatial distribution of dicrocoeliosis, and none in Asia. The present study was, therefore, undertaken to determine the prevalence and spatial distribution of dicrocoeliosis in the region and to describe the ecological niches that are favourable for the completion of the *Dicrocoelium* life cycle.

## Materials and Methods

### Study Areas

**Fig. 1:**
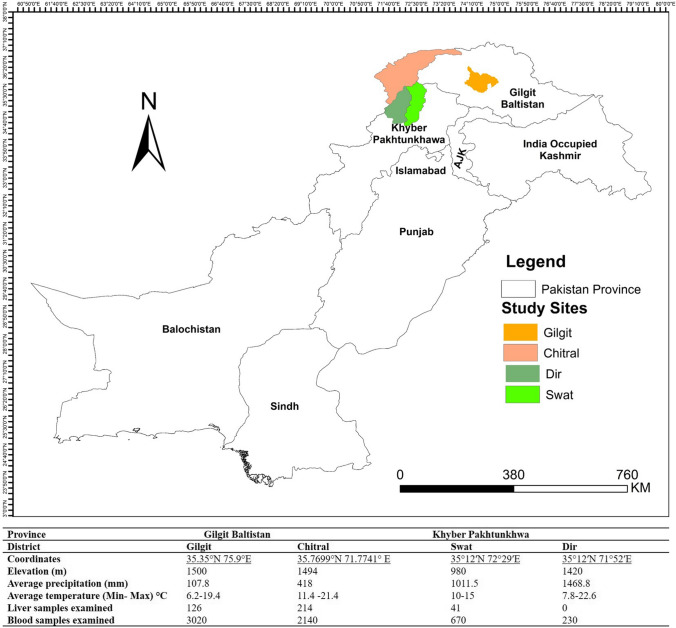
Locations of the Chitral, Gilgit, Swat and Dir study districts in the Himalayan ranges of Pakistan

The study area is comprised of the Gilgit Baltistan and Khyber Pakhtunkhwa provinces of Pakistan (Fig. 1). Gilgit Baltistan has a border with China through the Khunjerab pass, which occupies an area of over 72,971 km^2^. One district of Gilgit Baltistan was included in the study; (i) Gilgit district in the southwest of Karakoram range. The weather conditions include average rainfall of 120–240 mm annually. Additional irrigation is obtained from the rivers, which are abundant with melting snow water from higher altitudes. The Khyber Pakhtunkhwa has a border with Afghanistan to the west and north and spreads over an area of over 74,521 km^2^. Three districts of Khyber Pakhtunkhwa were included in the study; (ii) Chitral district to the north of the Indus river, which originates close to the holy mountain of Kailash in western Tibet. The average elevation is 1500 m and the daily mean temperature ranges from 4.1 °C to 15.6 °C, creating an arid environment with only patchy coniferous tree cover, and providing habitats that are hostile to many snail species; (iii) Swat district surrounded by Chitral and Dir districts. The area is predominantly rural, and most residents live in villages. The average elevation is 980 m, resulting in a considerably cool and wet climate with lush forests, verdant alpine meadows, and snow-capped mountains. The climate of the Swat district is warm and humid with short and moderate summers, temperature rarely rises above 37 °C. The annual rainfall averages around 33 inches with about 17 inches during June–September; (iv) Dir district borders to Afghanistan on the north and the Swat district to the east. The climate is cold, with average rainfall is 700 mm and the temperature varies from 6 °C to 38 °C.

### Study Design and Sample Collection

The study was carried out from July 2018 to September 2019. Random sampling was conducted and a total of 381 animals [Gilgit (*n* = 126), Chitral (*n* = 214), Swat (*n* = 41)] were examined for flukes recovery, animals belonging to 56 sheep flocks and 24 goat herds. The flukes were washed with phosphate-buffered saline (PBS) to remove adherent debris followed by Dicrocoelid morphological identification. A total of 6,060 blood samples [Gilgit (*n* = 3020), Chitral (*n* = 2140), Swat (*n* = 670) and Dir (*n* = 230)] were collected from 112 sheep and 48 goat herds. The blood samples were taken from the jugular vein of the animal herds and stored at 4 °C for 4–6 h before sera were separated. The number of blood samples to be collected was determined using the formula: $n\, = \,Z^{2} P \, \left( {1 - P} \right)/d^{2}$ [24], where *n* was the sample size, *Z* was the desired confidence interval (95%), *P* was a conservative estimate of the proportion of infected animals in the population (0.5) and *d* was precision of estimation or range in which the true population proportion is estimated to be (5%).

### Liver Sample Processing for Antigen Extraction

The liver samples were inspected for Dicrocoelid flukes to determine the infection rate among sheep and goats. Excretory/secretory (ES) and somatic antigens were extracted from Dicrocoelid flukes recovered from 33 positive liver samples as described by Gonzalez-Lanza, Manga-Gonzalez [25] with some modifications. Briefly, flukes were incubated in RPMI 1640 medium (Biosera, Boussens, France) supplemented with 200 mM N-acetyl-L-alanil-L-glutamine (Sigma), 0.3 g/l sodium bicarbonate 7.5% (Sigma) and 40 mg/l gentamycin at 37 °C for 48 h. After removal of the flukes, the medium was collected and centrifuged at 10,000 g for 15 min at 4 °C. To obtain a somatic extract, flukes were homogenised in tissue lysis buffer, and added according to the weight of tissue in a ratio of 1000 µl buffer/100 mg of tissue. The homogenate was then transferred to pre-chilled Eppendorf tubes and centrifuged at 10,000 rpm at 4 °C for 10 min. The supernatant was filtered through 0.22 μm pore size filter units and Protease Inhibitor Cocktail (P8340; Sigma) was added. Protein concentration was determined by the Bradford method [26]. Samples were aliquoted and stored at  – 80 °C until further processing.

### Enzyme-linked Immunosorbent Assay

ELISA was performed on 96-well microtiter plates as previously determined all incubation times by checkerboard titration method [27]. Briefly, each eluted antigen was mixed with coating buffer NaHCO3/Na2CO3 (Merck) in equal proportion (1:1) and 100 µl was added to each well of the microtiter plate and incubated overnight at 4 °C. The plates were washed three times with PBS containing 0.05% Tween 20 (Merck) and blocked with 0.05% BSA for 2 h at room temperature. 100 µl of the diluted sera from infected and control animals was added to each well and incubated for 2 h at 37 °C and washed three times with PBS containing 0.05% Tween 20. After washing, 100 µl/well goat anti-bovine IgG secondary antibodies (1: 10,000), conjugated with alkaline phosphatase (Invitrogen™ Cat. nos. WP20006, WP20007) were added and incubated for 1 h at room temperature. After washing the plates, 100 µl of the substrate para-nitrophenyl phosphate (PNPP) (Thermo Scientific™ Cat. No. 37621) was added and incubated at room temperature for 20 min. Finally, the reaction was stopped by the addition of 50 µl of 3 N NaOH solution, and the optical density (OD) value was recorded at 405 nm using an automated microplate reader. The sensitivity of the test was measured at 88% and the specificity was 95%, respectively (Supplementary Table S1). The sensitivity of the assay was determined using the formula: ${\text{Sensitivity}}\, = \,\left[ {a \, / \, \left( {a\, + \,c} \right)} \right]\, \times \,100$; where ‘*a*’ is the number of animals positive by ELISA and liver analysis (true positive), while ‘*c*’ is the number of animals positive by liver analysis but negative by ELISA (false negative). Similarly, ${\text{Specificity}}\, = \,\left[ {d \, / \, \left( {b\, + \,d} \right)} \right]\, \times \,100$; where ‘*d*’ is the number of animals negative by ELISA and liver analysis (true negative), while ‘*b*’ is the number of animals negative by liver analysis but positive by ELISA (false positive). The cut-off was calculated by the mean optical density (OD) of the negative reference serum, plus three times standard deviations (0.14 + 3*0.08 = 0.38). The cut-off value was set at 0.38, and sera with OD value higher or equal to 0.38 were considered positive.

### Species Distribution Models (SDMs)

Nineteen bioclimatic variables were obtained from the WorldClim (https://www.worldclim.org) global climate database (Fick and Hijmans, 2017) with the finest available resolution of approximately 1 km^2^. These layers were readable in ASCII format using ArcGIS 10.2 (ESRI, Redlands, CA, USA). The spatial patterns of *Dicrocoelium* infection were measured with MaxEnt based modelling with MaxEnt version 3.4.4 [28] Maxent is freely downloadable at http://www.cs.princeton.edu/~schapire/maxent/. Field visits were conducted to obtain the geographic coordinates of *Dicrocoelium*-infected animals, and Global Positioning System (GPS) location was used to obtain the precise coordinates of infected animal flocks and herds. If a flock or herd had multiple infected animals, only one point was recorded to avoid the spatial clusters of localities.

The occurrence data of *Dicrocoelium* based on liver and blood samples were filtered to reduce bias and to improve the performance of the ecological niches modelling. The SDM toolbox in ArGIS 10.2 software (ESRI, Redlands, CA, USA) was used to reduce the occurrence locations of each infected animal to a single point within 5 km. By eliminating duplicate occurrence points within the same pixel, *Dicrocoelium* presence points were reduced to 63 points from 160 presence points; 80% were used for the training and 20% for testing the model. 10,045 points were used to determine the MaxEnt distribution (background points and presence points). The model was run with the logistic output format where predicted values range from 0 (impossible) to 1 (optimal).

The performance of predicting the ecological niches of *Dicrocoelium* infection was evaluated using threshold-independent receiver operating characteristic (ROC) assessment, where the area under the ROC curve (AUC) was obtained for plotting the model’s sensitivity and specificity in MaxEnt. The geographical distribution of *Dicrocoelium* infection was mapped using a geographic information system (GIS). The presence points were marked on a world geodetic system (WGS84) reference coordinate system using high-resolution Google Earth and GIS coordinates. The parasite data were saved in an excel sheet and comma-separated values (CSV) files were used for the analysis. Compilation of geographic data and mapping was done by converting the excel data to the GIS format through Arc-Map (ESRI, Redlands, CA, USA).

To remove the autocorrelation among the 19 bioclimatic variables, Pearson’s correlation was used at (*r*^2^ ≥|0.8|) through the SDM Tools function in ArcGIS 10.2 (Universal tool; Explore climate data; Remove highly correlated variable). Five bioclimatic variables [Bio2 = mean diurnal range (mean of monthly (max temp—min temp), Bio4 = temperature seasonality (standard deviation × 100), Bio6 = min temperature of coldest month, Bio12 = annual precipitation and Bio15 = precipitation seasonality (coefficient of variation)] were used for the analysis. Additional variables with the same resolution as the bioclimatic variables were included in the evaluation; these were normalised difference vegetation index (NDVI) extracted from moderate resolution imaging spectroradiometer (MODIS) images, calculated from the visible and near-infrared light reflected by vegetation (NDVI data are available in Raster data images, each of which has several blocks which have specific values for different vegetation; and can be processed in a MaxEnt readable format using specific conversion tools), forest cover, elevation, derived from the digital elevation model (DEM) in ArcGIS 10.2, and distance to buildings or settlements. The environmental variables used in the MaxEnt model are summarised in Supplementary Table S2. The environmental variables associated with dicrocoeliosis were generated using a jacknife test in MaxEnt version 3.4.4 [28].

### Statistical Analysis

The relatedness of *Dicrocoelium* prevalence, based on blood and liver samples examination, with associated environmental and climatic risk factors, was calculated using Chi-squared test of independence in a statistical package for the social sciences (SPSS) version 20 (Armonk, NY: IBM Corp). The level of significance was set at *P* ≤ 0.05.

## Results

### Prevalence of *Dicrocoelium*

Overall, Dicrocoelid flukes were identified in 33 of 381 (8.66%) liver samples, and 238 of 6060 (3.93%) blood samples were positive for both *Dicrocoelium* IgG antibodies. *Dicrocoelium* was isolated from the liver samples of 20 of 56 sheep flocks and 13 of 24 goat herds*,* and blood samples showed the presence of *Dicrocoelium* IgG antibodies in 108 of 112 sheep flocks and 44 of 48 goat herds, respectively (Table 1). The seasonal percentage of *Dicrocoelium* positive liver samples was higher during the summer and autumn (10.88% and 10%, respectively) than during the winter and spring (5.22% and 6.9%, respectively); and a similar trend was seen in the blood samples, but neither of these seasonal differences was significant (*p* > 0.05). The percentage of *Dicrocoelium* positive blood samples was significantly higher (*p* = 0.0001) in females (4.93%) than in male hosts (1.47%), and a similar, but non-significant trend was seen in the liver samples. The percentage of *Dicrocoelium* positive blood samples was significantly higher (*p* = 0.05) in animals aged more than 3 years (4.5%) than in animals aged less than 1- year-old (3.26%), or 1 to 2 years old (3.33%). Similar, but non-significant trends were seen in the liver samples. The percentage of *Dicrocoelium* positive blood samples was significantly higher (*p* = 0.0001) in goats (7.39%) than in sheep (3.29%); while the percentage of *Dicrocoelium* positive liver samples was significantly higher (*p* = 0.0001) in sheep (10.04%) than in goats (5.74%). These data are shown in Table 2.

**Table 1:** Presence of *Dicrocoelium* in sheep and goat herds during the study period 2018–2019

		Liver samples					Blood samples				
Host	Breed	Total number of flocks/herds examined	*Dicrocoelium* positive flocks/herds in Chitral	*Dicrocoelium* positive flocks/herds in Gilgit	*Dicrocoelium* positive flocks/herds in Swat	*Dicrocoelium* positive flocks/herds in Dir	Total number of flocks/herds examined	*Dicrocoelium* positive flocks/herds in Chitral	*Dicrocoelium* positive flocks/herds in Gilgit	*Dicrocoelium* positive flocks/herds in Swat	*Dicrocoelium* positive flocks/herds in Dir
Sheep	Kelli	5	2	–	–	–	10	10	–	–	–
	Ramghani	9	5	–	–	–	18	18	–	–	–
	Balkhi	23	3	7	2	–	46	8	28	10	–
	Waziri	10	–	1	7	0	20	–	2	14	0
	Katchli	9	–	5	–	–	18	–	18	–	–
Total		56	10	13	9	0	112	36	48	24	0
Goat	Khurasani	13	7	5	–	0	26	14	6	–	2
	Cross Beetal	4	1	0	0	0	8	4	2	0	2
	Waziri	7	0	5	–	0	14	–	12	–	2
Total		24	8	10	0	0	48	18	20	0	6

**Table 2:** Prevalence of *Dicrocoelium* based on month, season, sex, age and host during the study period 2018–2019

Variables	Blood samples			Liver samples		
	Animals	Positive *n* (%)	*P* value	Animals	Positive *n* (%)	*P* value
Season						
Spring (March–April)	1020	30 (2.94)	$\chi 2=7.294$	29	2 (6.9)	$\chi 2=$ 2.96
Summer (May–Aug)	3070	116 (3.78)	p = 0.063^NS^	147	16 (10.88)	p = 0.398^NS^
Autumn (Sept–Oct)	1530	76 (4.97)		90	9 (10)	
Winter (Nov–Feb)	440	16 (3.64)		115	6 (5.22)	
Total	6060	238 (3.93)		381	33 (8.66)	
Sex						
Female	4296	212 (4.93)	$\chi 2=39$.69	222	22 (9.91)	$\chi 2=$ 1.05
Male	1764	26 (1.47)	p = 0.0001^**^	159	11 (6.92)	p = 0.306^NS^
Total	6060	238 (3.93)		381	33 (8.66)	
Age						
< 1 year	920	30 (3.26)	$\chi 2=5.718$	43	3 (6.98)	$\chi 2=4$.73
1–2 year	1984	66 (3.33)	p = 0.05^*^	152	8 (5.26)	p = 0.094^NS^
> 3 year	3156	142 (4.5)		186	22 (11.83)	
Total	6060	238 (3.93)		381	33 (8.66)	
Host						
Sheep	5113	168 (3.29)	$\chi 2=5536.3$	259	26 (10.04)	$\chi 2=349$.7
Goat	947	70 (7.39)	p = 0.0001^**^	122	7 (5.74)	p = 0.0001^**^
Total	6060	238 (3.93)		381	33 (8.66)	

### Geographical Distribution of *Dicrocoelium*

The prevalence of *Dicrocoelium* was highest in the Chitral district (7.1% and 9.81% positive blood and liver samples, respectively); followed by the Gilgit district (2.58% and 7.94% positive blood and liver samples, respectively); and lowest in Swat (1.19% and 4.88% positive blood and liver samples, respectively) and Dir (no positive samples, albeit the numbers of animals sampled in these districts, were small). Within each region, the prevalence of *Dicrocoelium* positive samples varied between different valleys from 0.5% (Doian valley in Gilgit) to 17.5% (Pret valley in Chitral) of blood samples and 3.85% (Torkhow valley in Chitral) to 18.18% (Raushan valley in Gilgit) of liver samples, as shown in Table 3. *Dicrocoelium* positive samples were identified in each valley in the Chitral and Swat districts. No *Dicrocoelium* positive samples were detected in the Barjangle, Singul and Bolan valleys in the Gilgit district; or in the Katair Dogdara and Maina Doag valleys of Dir district.

**Table 3:** Prevalence of *Dicrocoelium* in different localities of Gilgit, Chitral, Swat and Dir districts

Locations		Blood samples			Liver samples		
District	Valley	Number of animals	Number positive (%)	*P* value	Number of animals	Number positive (%)	*P* value
Gilgit							
	Phander	100	7 (7)	$\chi 2=$ 213.39	18	1 (5.56)	$\chi 2$= 19.064
	Dalomal	70	4 (5.71)	p = 0.0001^**^	24	4 (16.67)	p = 0.697^NS^
	Khonan Deh	70	5 (7.14)				
	Barsat	100	3 (3)				
	Yasin Valley	80	2 (2.5)		14	1 (7.14)	
	Damalgan	80	1 (1.25)		11	0	
	Sandhi	100	9 (9)				
	Raushan	95	7 (7.37)		11	2 (18.18)	
	Gahkuch	85	6 (7.06)				
	Barjangle	120	0				
	Singul	100	0				
	Rahim Abad	110	5 (4.55)				
	Chilmish Das	110	4 (3.64)				
	Danyor	100	4 (4)		7	0	
	Oshikhandas	80	3 (3.75)				
	Jaglot	150	2 (1.33)				
	Chalt Nagar	140	2 (1.43)		33	2 (6.06)	
	Chaprot	120	2 (1.67)				
	Hussain Abad	60	1 (1.67)				
	Rabat	50	1 (2)				
	Khizar Abad	140	2 (1.43)				
	Sikandar Abad	150	2 (1.33)				
	Jafar Abad	120	1 (0.83)				
	Harcho	90	1 (1.11)				
	Bunji	150	2 (1.33)				
	Doian	200	1 (0.5)				
	Gorikot	180	1 (0.56)		8	0	
	Bolan	70	0				
	Total	3020	78 (2.58)		126	10 (7.94)	
	Mean ± SEM	10.86 ± 6.82	2.79 ± 0.44 (3.25 ± 0.48)		15.75 ± 3.15	1.25 ± 0.49 (10.72 ± 2.18)	
Chitral							
	Booni	200	22 (11)		68	12 (17.65)	
	Mastuj	140	18 (12.86)		33	2 (6.06)	
	Chinar	110	3 (2.73)		19	2 (10.53)	
	Chuinj	110	3 (2.73)				
	Unshit	60	3 (5)		14	1 (7.14)	
	Shaidas	60	3 (5)				
	Gasht	80	4 (5)		7	1 (14.29)	
	Phargram	70	4 (5.71)				
	Phort	40	4 (10)				
	Lasht	40	3 (7.5)				
	Brock	50	5 (10)				
	Huzun	75	8 (10.67)				
	Balim	75	6 (8)				
	Raman	80	5 (6.25)				
	Harchin	80	6 (7.5)				
	Sor Laspor	100	4 (4)		17	1 (5.88)	
	Mori	33	2 (6.06)				
	Mori Payeen	27	1 (3.7)		7	0	
	Kaghozi	80	4 (5)				
	Singoor	80	4 (5)				
	Rondur	34	4 (11.76)		5	0	
	Riri Qwir	26	2 (7.69)				
	Barenis	50	7 (14)				
	Pret	40	7 (17.5)				
	Kiyar	30	2 (6.67)				
	Drosh	88	3 (3.41)		4	0	
	Brun	82	4 (4.88)		8	1 (12.5)	
	Garam Chashma	60	3 (5)		6	0	
	Torkhow	140	8 (5.71)		26	1 (3.85)	
Total	2140	152 (7.1)		214	21 (9.81)		
Mean ± SEM	73.79 ± 7.3	5.24 ± 0.83 (7.25 ± 0.67)		17.83 ± 5.26	1.75 ± 0.95 (9.74 ± 1.39)		
Swat							
	Bankhwar	130	2 (1.54)				
	Gabral	110	2 (1.82)		11	1 (9.09)	
	Utrar	130	1 (0.77)		6	0	
	Kalam	90	1 (1.11)		15	0	
	Boyun	130	1 (0.77)		9	1 (11.11)	
	Matiltan	80	1 (1.25)				
	Total	670	8 (1.19)		41	2 (4.88)	
	Mean ± SEM	111.67 ± 9.1	1.33 ± 0.21 (1.21 ± 0.17)		10.25 ± 1.89	0.5 ± 0.29 (10.1 ± 0.71)	
Dir							
	Katair Dogdara	120	0				
	Maina Doag	110	0				
	Total	230	0		0	0	
	Mean ± SEM	115 ± 5	0		0	0	
Overall		6060	238 (3.93)		381	33 (8.66)	

### Spatial Patterns of *Dicrocoelium* Infection

**Fig. 2:**
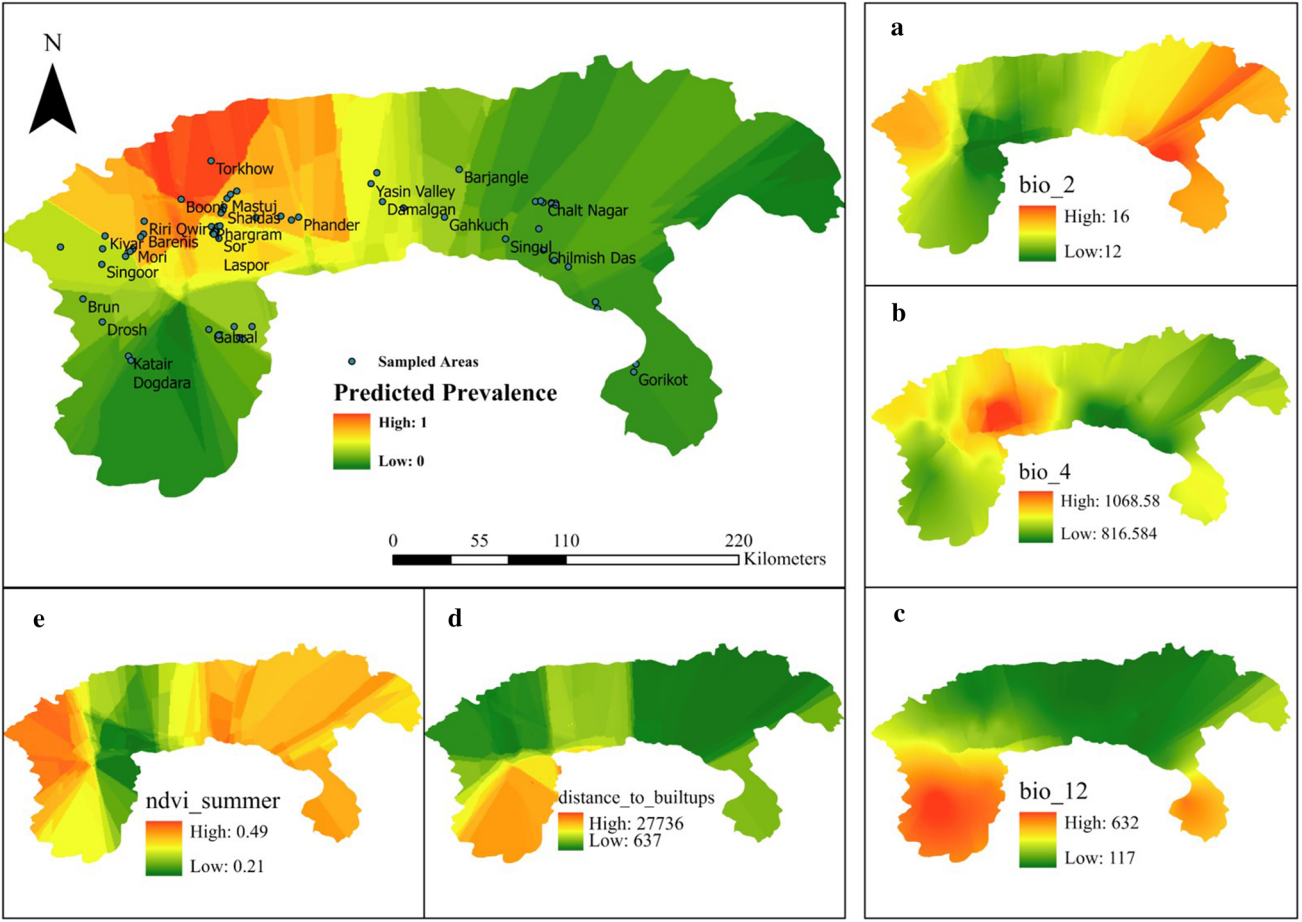
Predicted spatial pattern based on blood and liver sample results of ecological niches predicted to support *Dicrocoelium* infection from 2018 to 2019. Red shading indicates the most suitable niches for Dicrocoelid flukes, and green shading predicts the least suitable conditions. The MaxEnt model predictions for the contributions of variables to the occurrence of dicrocoeliosis are shown in **a** (mean monthly diurnal temperature range), **b** (temperature seasonality), **c** (annual precipitation), **d** (distance from built-up areas) and **e** (normalised difference vegetation index)

The map based on the *Dicrocoelium* occurrence of positive samples predicted the most likely ecological niches to support *Dicrocoelium* infection to be in the central parts of Chitral, extending towards the upper and lower Chitral districts (Fig. 2). Although *Dicrocoelium* infection was identified from parts of Gilgit, and areas of Swat and Dir bordering Chitral, MaxEnt modelling predicted lower risk of *Dicrocoelium* occurrence in these overall study regions.

The MaxEnt model predicted that the two climatic variables of the mean diurnal temperature range (Bio2) and temperature seasonality (Bio4) contributed most to the occurrence of dicrocoeliosis in the Gilgit and lower and upper parts of the Chitral (Fig. 2a,b). However, annual precipitation (Bio 12) and distance to built-up areas were predicted to contribute most to the occurrence of dicrocoeliosis in upper Dir and Sawat districts (Fig. 2c,d); while summer NDVI values predicted *Dicrocoelium* active zones in the upper Dir and lower Chitral districts (Fig. 2e).

### Contribution of Ecological Niches and Climatic Variables on *Dicrocoelium* Infection

The AUC values for the training and test data were 0.987 and 0.985, respectively, suggesting an excellent predictive power for the model (Supplementary Fig. S1). The results of jackknife analysis performed on five climatic and four geographical variables are shown in Supplementary Figure S2. Cross comparison of these nine variables in MaxEnt revealed that only four, namely annual precipitation (10.4%), mean diurnal range (mean of monthly max temp-min temp) (7.7%), distance from population built-up areas (9.1%) and vegetation index in spring (56.7%), were effective and would have contributed most to the model development. The six most influential variables observed in the present study are shown in Supplementary Fig. S3. The result shows that the occurrence of *Dicrocoelium* infection was directly related to the mean of the monthly diurnal temperature range (Bio2), temperature seasonality (Bio4), mean temperature of the coldest month (Bio6), distance from population built-up areas and summer NDVI. An inverse relationship was observed between annual precipitation (Bio12) and the identification of *Dicrocoelium* infection.

## Discussion

In the present study, 381 liver samples and 6060 blood samples provide a valuable resource which can be used to describe aspects of the epidemiology of dicrocoeliosis in the Himalayan ranges of Pakistan. The estimated prevalence of dicroceliosis in sheep and goats in the Gilgit and Chitral districts was higher than reported incomparable Asian studies conducted in India [29], Iran [30], and Iraq [31]. While direct comparisons are biased by differences in study design, the relatively high prevalence confirms the widespread nature of ecological niches that can support the continuity of the *Dicrocoelium* life cycle in the northwest of Pakistan. Characteristics including calcium-rich, alkaline soils and diverse vegetation help to provide overlapping niches that are suited to each of the intermediate and definitive hosts [4]. The prevalence of *Dicrocoelium* was highest during the summer and autumn, as previously described in Algerian cattle (Chougar, Harhoura [32], but the seasonal differences were not significant, and potentially may have been confounded by factors such as the age, species and breed of the animals and sampling location. The suitability of environmental factors for the development and growth of intermediate snails and ant hosts and grazing patterns enabling exposure to metacercaria-infected ants [4] will vary throughout the year. However, in the absence of effective anthelmintic treatments for dicrocoeliosis [5], animals accumulate infections acquired during different periods throughout their lives; consequently, a cross-sectional study involving animals more than 1-year-old cannot identify seasonal infection risks. Extreme cold weather conditions in the Himalayan ranges of Pakistan preclude grazing of animals on open pastures during the winter months and imply that the greatest risk of infection is during the spring and summer when conditions are also favourable for intermediate host development [33].

The estimated prevalence of *Dicrocoelium* was higher in female hosts and highest in animals aged more than 3 years. Previous reports have shown higher prevalences in female hosts [32, 34] and suggested a relationship between periparturient susceptibility due to pregnancy and lactation stress [35]. However, female animals are more likely to be retained for breeding, hence live for longer and have more opportunities to become infected with *Dicrocoelium*. The specie prevalence could be explained by the possibility of higher susceptibility of sheep than goats. Higher prevalence and worm burden in sheep could be the result of more sensitive species, but goats have contact "infection" with *Dicrocoelium*, but this does not go advance. This could explain the higher prevalence of antibodies, but not found in adults. The different results further highlight challenges of sample size and diagnosis of adults could be less sensitive, with a high number of false negatives in goats than in sheep. It has been suggested that browsing goats are less likely to be infected than grazing sheep [34], albeit *Dicrocoelium*-infected ants may migrate high enough onto herbage to be ingested by browsing animals. However, the ecological information on ants and land snails involved as intermediate hosts in these areas is still unknown.

The highest occurrence of *Dicrocoelium* infection was recorded in the Chitral district, consistent with its high altitude pastureland fed by melting of glacier water and high seasonal rainfall providing the most suitable conditions for completion of the parasite’s life history. A similar situation has been described in Spain, where *Dicrocoelium* infection is most frequent in areas with high altitudes, lower winter temperatures and high rainfall [36]. The occurrence of *Dicrocoelium* infection in the Gilgit, Swat and Dir districts was moderate to low associated with lower rainfall and more humid environments.

Prediction of the environmental suitability and geographical distribution of ecological niches, climatic and anthropomorphic factors that are suited to the completion of the *Dicrocoelium* life cycle is needed to inform strategic disease control. SDMs have been used to predict the special distribution of *Dicrocoelium* infection in Iran [19] and Spain [36]. The ROC test showed a high validity of the SDM in predicting favourable ecological niches for these parasites in the Himalayan ranges of Pakistan. The MaxEnt model revealed that the most influential climatic variables associated with a positive effect on the risk of dicrocoeliosis were the mean of the monthly diurnal temperature range (Bio2), temperature seasonality (Bio4) and the mean temperature of the coldest month (Bio6); while an inverse relationship was observed for annual precipitation (Bio12). The results suggest that these factors play a key role in the development, survival and transmission of Dicrocoelid flukes and their intermediate hosts. The results also found a high correlation between distance from population built-up areas and summer NDVI and the presence of *Dicrocoelium* infection, explained by the observation that forest areas with permanent pastures, good water availability and suitable soil type provide suitable habitats for land snails and ant intermediate hosts, and opportunities for final host infection [37].

Overall this study shows a high estimated prevalence of dicrocoeliosis in the Himalayan ranges of Pakistan. The ecological niche model helps to describe factors that increase the risk of infection, providing information that might help in the development of targeted evasive management strategies and in predicting the potential spread of *Dicrocoelium* to other suitable habitats in the region.

## Conclusion

In the present study, the diagnosis of dicrocoeliosis was based both on the identification of Dicrocoelid flukes in the livers of slaughtered animals and on positive blood sample results using a bespoke combination of ES and somatic antigen ELISAs. The random sampling methods that were used to collect the diagnostic samples helped describe the spatial distribution of *Dicrocoelium* infection and provided a crude estimation of the parasite’s prevalence. However, the fold difference in overall prevalence estimates obtained from the liver (~ 9%) and blood (~ 4%) sample results highlight important difficulties in the accurate determination of the prevalence of fluke parasites; namely the adequacy of the sample size, precise knowledge of the sensitivities and specificities of the diagnostic tests used, and the representativeness of the study populations. In the current study, the blood sample size was adequate, but the number of liver samples was too low to allow for precise analysis; the true sensitivities and specificities of the diagnostic tests were unknown; and the live and slaughtered animal populations may have differed in their origins, grazing management, and are known to differ in demographic characteristics such as sex, age, species and breed. The number of samples that could be collected and processed was constrained by the remoteness and poor supporting infrastructure of the study region. Nevertheless, the 381 liver samples and 6060 blood samples provide a valuable resource which can be used to describe aspects of the epidemiology of dicrocoeliosis in the Himalayan ranges of Pakistan. In the absence of a gold standard, the accurate determination of the sensitivities and specificities of diagnostic tests for the study of fluke parasite epidemiology is challenging [38], and requires different samples to be collected from the same animals in a manner which was not feasible in the current study.

## Supplementary Information

Below is the link to the electronic supplementary material.

Supplementary file1 (JPG 64 KB) Fig. S1 ROC curve calculated by MaxEnt plotting average sensitivity against 1 - specificity for prediction of Dicrocoelium

Supplementary file2 (JPG 105 KB) Fig. S2 Jackknife test of regularised training gain of variables examined in the Dicrocoelium habitat suitability model. Blue bars represent the gain when the environmental variable is used in isolation; green bars represent the gain when the environmental variable is omitted; the red bar represents the gain when using all of the environmental variables

Supplementary file3 (JPG 289 KB) Fig. S3 The response curves for suitable variables were obtained by the logistic output format for mean diurnal temperature range (bio2), seasonal temperature variation (bio4), mean temperature of the coldest month (bio6), annual precipitation (bio12), distance to build-up areas, and summer normalised digital vegetation index

Supplementary file4 (DOCX 14 KB)

Supplementary file5 (DOCX 16 KB)

## Data Availability

Maxent software is freely downloadable at http://www.cs.princeton.edu/~schapire/maxent/. The data files used to calculate the model are available on request.
