# Supplementary figures and images for: Spatial Distribution of Dicrocoelium in the Himalayan Ranges: Potential Impacts of Ecological Niches and Climatic Variables
Source: Acta Parasitol. 2022 Nov 22;68(1):91–102. doi: 10.1007/s11686-022-00634-1 (PMC10011340; doi:10.1007/s11686-022-00634-1)

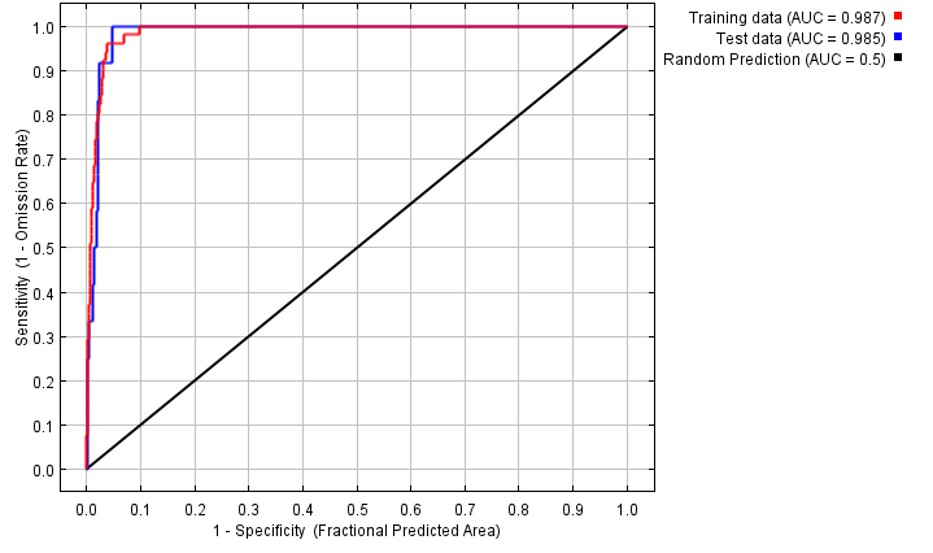

Supplement: Supplementary file 1 — Supplementary file1 (JPG 64 KB) Fig. S1 ROC curve calculated by MaxEnt plotting average sensitivity against 1 - specificity for prediction of Dicrocoelium [file 11686_2022_634_MOESM1_ESM.jpg]

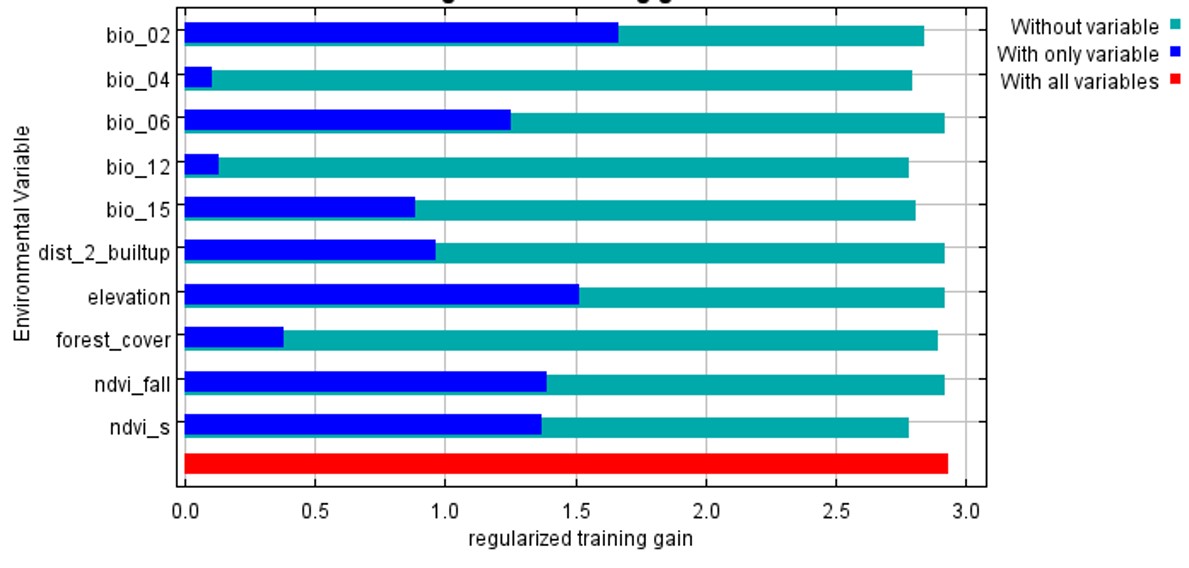

Supplement: Supplementary file 2 — Supplementary file2 (JPG 105 KB) Fig. S2 Jackknife test of regularised training gain of variables examined in the Dicrocoelium habitat suitability model. Blue bars represent the gain when the environmental variable is used in isolation; green bars represent the gain when the environmental variable is omitted; the red bar represents the gain when using all of the environmental variables [file 11686_2022_634_MOESM2_ESM.jpg]

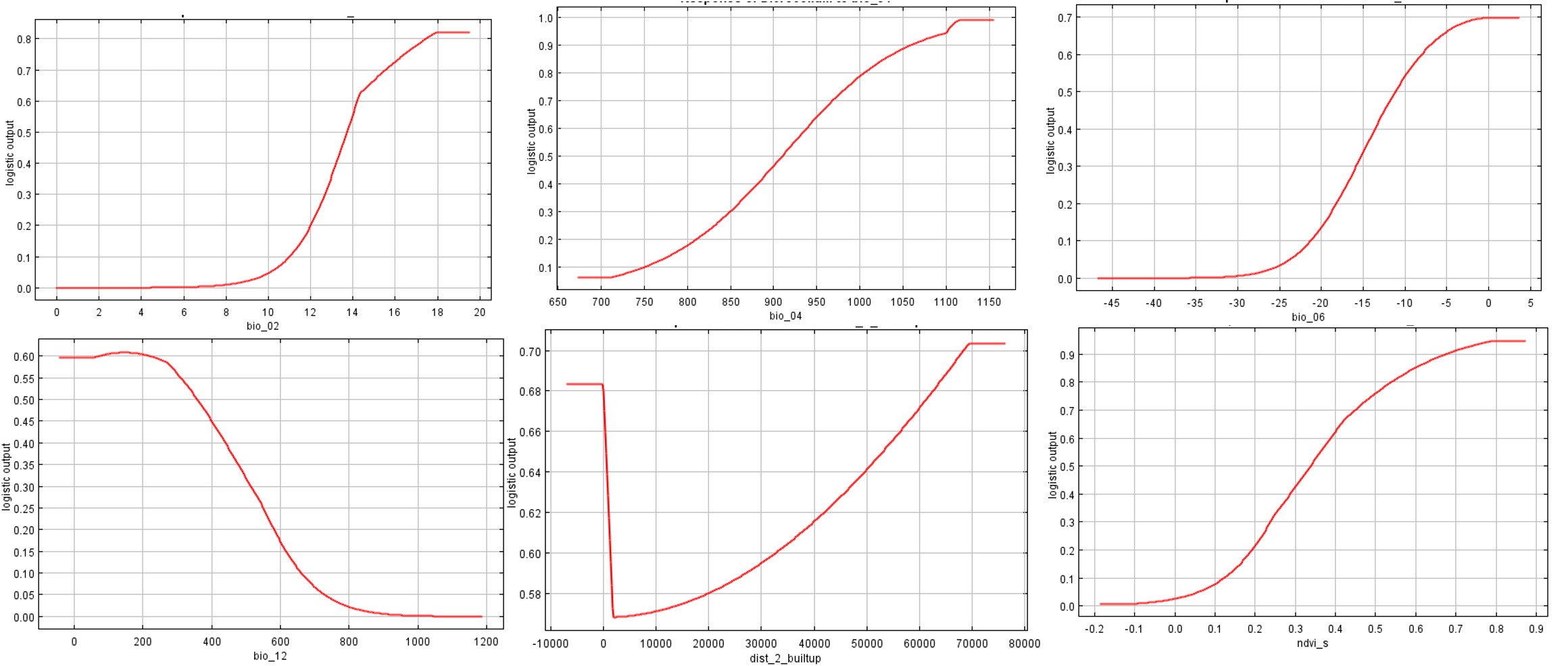

Supplement: Supplementary file 3 — Supplementary file3 (JPG 289 KB) Fig. S3 The response curves for suitable variables were obtained by the logistic output format for mean diurnal temperature range (bio2), seasonal temperature variation (bio4), mean temperature of the coldest month (bio6), annual precipitation (bio12), distance to build-up areas, and summer normalised digital vegetation index [file 11686_2022_634_MOESM3_ESM.jpg]
