# Supplementary material for: Spatial Distribution of Dicrocoelium in the Himalayan Ranges: Potential Impacts of Ecological Niches and Climatic Variables
Source: Acta Parasitol. 2022 Nov 22;68(1):91–102. doi: 10.1007/s11686-022-00634-1 (PMC10011340; doi:10.1007/s11686-022-00634-1)
Supplement: Supplementary file 4 — Supplementary file4 (DOCX 14 KB) [file 11686_2022_634_MOESM4_ESM.docx]

**Supplementary Table S1:** Diagnostic efficacy of ELISA established for ES/somatic antigens.

| **Test** | **ELISA Test** | | | **Sensitivity** | **Specificity** | **KAPPA** |
| --- | --- | --- | --- | --- | --- | --- |
| **Liver examination** | **Positive** | **Negative** | **Total** | **95% CI** | **95% CI** |  |
| Positive | 29 | 4 | 33 | 87.9% (71.8- 96.6) | 94.6% (81.8-99.3) | Kappa= 0.823 |
| Negative | 2 | 33 | 35 |  |  | SE of kappa = 0.069 |
|  |  |  | 68 |  |  | 95% confidence interval: 0.688 to 0.958 |
