# Supplementary material for: Spatial Distribution of Dicrocoelium in the Himalayan Ranges: Potential Impacts of Ecological Niches and Climatic Variables
Source: Acta Parasitol. 2022 Nov 22;68(1):91–102. doi: 10.1007/s11686-022-00634-1 (PMC10011340; doi:10.1007/s11686-022-00634-1)
Supplement: Supplementary file 5 — Supplementary file5 (DOCX 16 KB) [file 11686_2022_634_MOESM5_ESM.docx]

**Supplementary Table S2:** List of environmental variables used in MaxEnt model

| **Environmental variables** | | **Interpretation** | **Source** |
| --- | --- | --- | --- |
| Bio1 | Annual mean temperature (°C) | | [http://www.worldclim.org](http://www.worldclim.org/) |
| Bio2 | Mean diurnal range [mean of monthly  (max temp - min temp)] (°C) | | [http://www.worldclim.org](http://www.worldclim.org/) |
| Bio3 | Isothermality (Bio2/Bio7) (x100) | | [http://www.worldclim.org](http://www.worldclim.org/) |
| Bio4 | Temperature seasonality (standard deviation x100) | | [http://www.worldclim.org](http://www.worldclim.org/) |
| Bio5 | Max temperature of warmest month (°C) | | [http://www.worldclim.org](http://www.worldclim.org/) |
| Bio6 | Minimum temperature of coldest month (°C) | | [http://www.worldclim.org](http://www.worldclim.org/) |
| Bio7 | Temperature annual range (Bio5-Bio6) (°C) | | [http://www.worldclim.org](http://www.worldclim.org/) |
| Bio8 | Mean temperature of wettest quarter (°C) | | [http://www.worldclim.org](http://www.worldclim.org/) |
| Bio9 | Mean temperature of driest quarter (°C) | | [http://www.worldclim.org](http://www.worldclim.org/) |
| Bio10 | Mean temperature of warmest quarter (°C) | | [http://www.worldclim.org](http://www.worldclim.org/) |
| Bio11 | Mean temperature of coldest quarter (°C) | | [http://www.worldclim.org](http://www.worldclim.org/) |
| Bio12 | Annual precipitation (mm) | | [http://www.worldclim.org](http://www.worldclim.org/) |
| Bio13 | Precipitation of wettest month (mm) | | [http://www.worldclim.org](http://www.worldclim.org/) |
| Bio14 | Precipitation of driest month (mm) | | [http://www.worldclim.org](http://www.worldclim.org/) |
| Bio15 | Precipitation seasonality (coefﬁcient of variation) | | [http://www.worldclim.org](http://www.worldclim.org/) |
| Bio16 | Precipitation of wettest quarter (mm) | | [http://www.worldclim.org](http://www.worldclim.org/) |
| Bio17 | Precipitation of driest quarter (mm) | | [http://www.worldclim.org](http://www.worldclim.org/) |
| Bio18 | Precipitation of warmest quarter (mm) | | [http://www.worldclim.org](http://www.worldclim.org/) |
| Bio19 | Precipitation of coldest quarter (mm) | | [http://www.worldclim.org](http://www.worldclim.org/) |
| NDVI | | Normalized difference vegetation index | NASA: http://modis-land.gsfc.nasa.gov/vi.html |
| Elevation | | elevation of the areas (m) | Derived in ArcGIS 10.2 from DEM |
| forest cover | | Type of forest |  |
| distance to buildings/settlements | | Density of settlements (m) | Calculated in Arc GIS 10.2 |

Abbreviations: mm millimeter; °C degree Celsius; Bio bioclimatic variables; max maximum; min minimum
